# Draft Genome Sequence of Acinetobacter calcoaceticus TUS-SO1, a Bacterium Capable of Degrading 2-Phenoxyacetophenone

**DOI:** 10.1128/mra.00165-23

**Published:** 2023-05-24

**Authors:** Hirari Nakagawa, Hiroki Kaneko, Toshiki Furuya

**Affiliations:** a Department of Applied Biological Science, Faculty of Science and Technology, Tokyo University of Science, Chiba, Japan; University of Arizona

## Abstract

Acinetobacter calcoaceticus TUS-SO1 degrades 2-phenoxyacetophenone, a model compound for the β-*O*-4 linkage in lignin. Here, we report the whole-genome sequence of this bacterium. The draft genome comprises 4,284,351 nucleotides and 3,976 coding DNA sequences, with an average G+C content of 38.5%.

## ANNOUNCEMENT

Lignin-degrading microorganisms play an important role in the natural global carbon cycle. Lignin is a heterogeneous aromatic polymer that forms plant cell walls. Microorganisms that degrade lignin-related compounds are useful for the production of various industrial chemicals, such as aromatic compounds (1–3). A bacterial strain involved in lignin degradation and belonging to the genus Acinetobacter, TUS-SO1, was recently isolated from soil in Chiba, Japan (4). Strain TUS-SO1 can degrade 2-phenoxyacetophenone (2-PAP), a model compound for the β-*O*-4 linkage in lignin. Strain TUS-SO1 oxidatively and selectively cleaves the ether bond of 2-PAP to produce phenol and benzoate (4). The genome of this bacterium was sequenced to expand our understanding of the ether bond–cleaving ability and lignin-degrading potential of Acinetobacter.

Strain TUS-SO1 was cultured in LB medium, consisting of (per liter) tryptone (10 g), Bacto yeast extract (5 g), and NaCl (10 g) (pH 7.2). A single colony was picked and inoculated into this medium. The strain was cultured at 30°C for 24 h. Bacterial genomic DNA was extracted using a NucleoBond high-molecular-weight (HMW) DNA kit (Macherey-Nagel, Düren, Germany) according to the manufacturer’s recommendations. All genomic DNA samples were sequenced by the Taniguchi Dental Clinic Oral Microbiome Center (Kagawa, Japan), following the standard workflow for library preparation. For short-read sequencing, genomic libraries were prepared using an MGIEasy FS DNA library prep set (MGI, Shenzhen, China), and sequencing was performed using a DNBSEQ-G400FAST sequencer and DNBSEQ-G400RS high-throughput rapid sequencing set (2 × 150 bp; MGI).

A total of 60,144,228 reads with a paired-end read length of 150 bp were obtained. The reads were initially assessed using FastQC v0.11.9 (5). Adapters and low-quality reads were trimmed using Fastp v0.20.1, and read pairs were collected from the adapter-trimmed sequences using SeqKit v2.2.0 (6, 7). A total of 3,009,254 trimmed reads were utilized for *de novo* assembly using Platanus_B v1.3.2 (8). Gene annotation was carried out using DFAST v1.6.0 (9). CheckM was used to check the completeness of the draft genome sequence in DFAST v1.6.0 (10). Taxonomic positions were determined using the digital DNA-DNA hybridization (dDDH) method (formula *d*_4_) (11) with the Type (Strain) Genome Server (TYGS) v358 (12, 13). The average nucleotide identities (ANIs) with currently available Acinetobacter sp. genome sequences were calculated using OAT v0.93 (14). A phylogenomic tree was inferred from genome BLAST distance phylogeny (GBDP) distances, determined using the TYGS pipeline (12, 13). The tree was visualized and modified for publication using iTOL (15). Default parameters were used for all software analyses unless otherwise specified.

**FIG 1:**
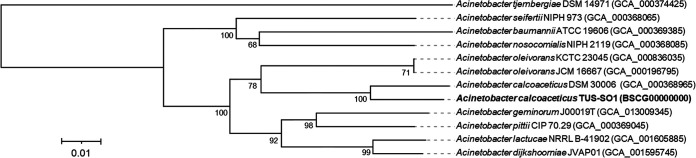
Phylogenomic tree of strain TUS-SO1 and type strains of the genus Acinetobacter available from the TYGS database. Branch lengths are scaled in terms of GBDP distance formula *d*_5_, and numbers above the branches indicate GBDP pseudobootstrap support values of >60% from 100 replications, with an average branch support of 89.6%; the tree was rooted at the midpoint.

The draft genome contained 75 contigs spanning 4,284,351 bp, with a coverage of 105×, mean G+C content of 38.5%, *N*_50_ value of 386,117 bp, and total of 3,976 genes. The genome encoded a total of 13 rRNA and 64 tRNA genes. CheckM analysis showed 100.0% completeness and 0.15% estimated contamination. A TYGS analysis indicated that its sequence had 68.1% dDDH (95% confidence interval, 65.1% to 71.0%) with respect to that of A. calcoaceticus DSM 30006^T^. Strain TUS-SO1 showed an ANI of 96.20% to A. calcoaceticus DSM 30006^T^. The phylogenomic tree also showed that TUS-SO1 is closely related to A. calcoaceticus (Fig. 1). The draft genome sequence of strain TUS-SO1 provides valuable genetic information that will enhance our understanding of the ether bond–cleaving ability and lignin-degrading potential of A. calcoaceticus.

### Data availability.

The BioProject, BioSample, and DRA/SRA accession numbers for the sequence reported here are PRJDB14715, SAMD00554158, and DRR415795, respectively. The GenBank accession number of the deposited genome is BSCG00000000.
